# cAMP signaling factors regulate carbon catabolite repression of hemicellulase genes in *Aspergillus nidulans*

**DOI:** 10.1186/s13568-022-01467-x

**Published:** 2022-10-01

**Authors:** Emi Kunitake, Ryota Uchida, Keisuke Asano, Kyoko Kanamaru, Makoto Kimura, Tetsuya Kimura, Tetsuo Kobayashi

**Affiliations:** 1 Department of Life Sciences, Graduate School of Bioresources, Mie University, 1577 Kurimamachiya-Cho, Tsu, Mie 514-8507 Japan; 2 Department of Applied Biosciences, Graduate School of Bioagricultural Sciences, Nagoya University, Furo-Cho, Chikusa-Ku, Nagoya, Aichi 464-8601 Japan; 3 Present Address: Department of Biological Chemistry, Chubu University, 1200 Matsumoto-Cho, Kasugai, Aichi 487-8501 Japan

**Keywords:** *Aspergillus nidulans*, Carbon catabolite repression, Xylanase, Mannanase, cAMP-dependent protein kinase

## Abstract

Carbon catabolite repression (CCR) enables preferential utilization of easily metabolizable carbon sources, implying the presence of mechanisms to ensure discriminatory gene repression depending on the ambient carbon sources. However, the mechanisms for such hierarchical repression are not precisely understood. In this report, we examined how deletion of *pkaA* and *ganB*, which encode cAMP signaling factors, and *creA*, which encodes a well-characterized repressor of CCR, affects CCR of hemicellulase genes in the filamentous fungus *Aspergillus nidulans*. β-Xylanase production increased not only in Δ*creA* but also in Δ*pkaA* and Δ*ganB*, with the highest level observed in their double deletants, irrespective of the presence or absence of d-glucose. Expression of the β-xylanase genes in the presence of d-glucose was de-repressed in all the deletion mutants, with significantly higher tolerance against d-glucose repression in Δ*pkaA* and Δ*ganB* than in Δ*creA*. In the presence of galactomannan and d-glucose, partial de-repression of β-mannanase production was detected in Δ*creA*, but not in Δ*pkaA* and Δ*ganB*. The double deletion of *creA*/*pkaA* and *creA*/*ganB* led to earlier production. Release from d-glucose repression of the β-mannanase genes was partial in the single deletants, while nearly full de-repression was observed in Δ*creA*Δ*pkaA* and Δ*creA*Δ*ganB*. The contribution of PkaA and GanB to CCR by d-xylose of the β-mannanase genes was very minor compared to that of CreA. Consequently, the present study revealed that cAMP signaling plays a major role in CCR of hemicellulase gene expression in a manner that is clearly independent from CreA.

**Supplementary Information:**

The online version contains supplementary material available at 10.1186/s13568-022-01467-x.

## Introduction

Plant cell walls account for the largest amount of biomass on Earth and are an important material as an alternative to fossil resources. They consist of various high-molecular-weight components including polysaccharides such as cellulose, which is their main component, and hemicelluloses such as β-xylan and β-mannan. Efficient enzymatic hydrolysis of hemicellulose is as important for biorefining as the degradation of cellulose. Saprophytic filamentous fungi such as *Aspergillus* are important as a source of enzymes for biomass degradation, since the fungi produce a variety of cellulases and hemicellulases. The genes encoding these enzymes are generally transcriptionally regulated in response to inducing carbon sources as well as repressing ones, but the molecular mechanisms of regulation are still not fully understood.

Carbon catabolite repression (CCR) is a mechanism that acts in the presence of easily metabolizable carbon sources such as d-glucose and causes the repression of genes involved in the utilization of alternative carbon sources. In filamentous fungi, the expressions of polysaccharide-degrading enzyme genes encoding α-amylase, cellulase, and hemicellulase, as well as other enzymatic genes required for the utilization of carbon sources, are under the control of CCR. CCR in *Aspergillus* and related genera involves the C_2_H_2_-type transcription factor CreA or its orthologs that repress gene expression by binding directly to the SYGGRG motif present in the promoters of the target genes (Cubero and Scazzocchio 1994; Kulmburg et al. 1993). The activity of CreA is regulated by post-translational phosphorylation, which affects subcellular localization, stability, and DNA binding (Alam et al. 2017; de Assis et al. 2021).

The cAMP signaling pathway is a conserved signal transduction system among eukaryotes. In *Aspergillus nidulans*, it regulates conidial germination, vegetative growth, asexual development, stress response, and secondary metabolism (Fillinger et al. 2002; Krijgsheld et al. 2013; Ni et al. 2005; Shimizu and Keller 2001). It is also involved in CreA-dependent CCR, since cAMP-dependent protein kinase (PkaA in *A. nidulans*) activity affects the subcellular localization, phosphorylation, and stability of CreA (de Assis et al. 2015, 2020, 2021; Ribeiro et al. 2019). Furthermore, cAMP signaling also regulates CreA-independent CCR of cellulase gene expression (Kunitake et al. 2019), in which PkaA and one of the trimeric G-protein alpha subunits (GanB) have been shown to be involved. CreA-independent CCR is triggered by various monosaccharides, especially hexoses such as d-glucose and d-mannose. In the case of pentoses such as d-xylose, CreA-dependent CCR acts as the predominant mechanism.

To extend knowledge on the CreA-independent CCR with special interest in its target enzymes other than cellulases, we focused on β-xylanases and β-mannanases because xylan and mannan are major polysaccharides next to cellulose in lignocellulosic materials and also because the enzymes are important industrial enzymes with high demand. β-Xylanase genes *xlnA* and *xlnB* are repressed by d-glucose via CreA-dependent CCR in *A. nidulans* (Orejas et al. 1999, 2001). The gene for the β-xylanase gene-specific transcriptional activator XlnR is also repressed in a CreA-dependent manner (Tamayo et al. 2008). However, it remains to be clarified whether CreA-independent CCR regulates β-xylanase genes. In addition, there is little information on CCR of β-mannanase genes, which encode another hemicellulase important for industrial use. In this study, the effects of *creA*, *pkaA*, and *ganB* deletion on CCR of β-xylanase and β-mannanase genes were compared to clarify whether CreA-independent CCR participates in their regulation, with a special interest in regulation by cAMP signaling.

## Materials and methods

### Strains and growth conditions

The *A. nidulans* strains used in this study were constructed in a previous study and are listed in Additional file 1: Table S1 (Kunitake et al. 2019). ABU was used as a reference strain. Strains except for Δ*pkaA* and Δ*creA*Δ*pkaA* were grown at 37 °C in standard minimal medium (MM) (Rowlands and Turner 1973). l-arginine (0.525 g/L), d-biotin (0.02 mg/L), pyridoxine–HCl (0.5 mg/L), or uridine (1.0 g/L) were added depending on the auxotrophy of the strains. The strains Δ*pkaA* and Δ*creA*Δ*pkaA* were propagated on MAG medium containing 0.5 M NaCl, which has been reported to remediate the growth of Δ*pkaA* (De Souza et al. 2013).

### Enzyme assays

*A. nidulans* strains were precultured in MM containing 1% Bacto™ Peptone (Becton Dickinson, Franklin Lakes, NJ, USA) and 0.1% Bacto™ Yeast Extract (Becton Dickinson) instead of d-glucose for 22 h at 37 °C, harvested by filtration, and washed with MM without carbon sources. Then the harvested mycelia of the same wet weight (0.01 g/mL culture) were transferred into fresh MM containing 1% d-xylose or 0.5% Locust Bean Gum (LBG) (Sigma-Aldrich, MO, USA) to induce β-xylanase and β-mannanase production, respectively. d-Glucose was added as the repressing carbon source at a final concentration of 1%. β-Xylanase activity in the culture filtrates was measured using Azo-Xylan from Birchwood (Megazyme, Ireland) as a substrate. Enzyme reactions were carried out by referring to the manufacturers’ instructions and a report by Tamayo et al. (Tamayo et al. 2008). Reaction mixtures containing 1% substrate in 0.1 M succinate buffer (pH 5.5) and culture supernatant were incubated at 40 °C for 10 min and then stopped by the addition of 95% ethanol. Precipitated nondegraded substrate was removed by centrifugation. Absorbance at 595 nm for the remaining supernatants was measured using a Model 680 microplate reader (Bio-Rad, CA, USA). β-Mannanase activity was also measured as above, except that Azo-Carob Galactomannan (Megazyme) was used as the substrate. One unit of enzyme activity was defined as the amount of enzyme that increases the absorbance by 0.1 per minute. The weight of dry mycelia was measured after drying at 100 °C for 48 h to evaluate fungal growth.

### Transcriptional analysis

Precultured and washed *A. nidulans* mycelia as described for the enzyme assays were transferred into MM containing 3 mM d-xylose or 3 mM β-mannobiose (Megazyme) with or without repressing carbon sources and incubated for 1.5 h at 37 °C. d-Glucose or d-xylose at 30 mM were used as the repressing carbon sources. After incubation, mycelia were harvested and frozen in liquid nitrogen. The resulting frozen mycelia were physically broken with an SK-100 (Tokken, Japan) to obtain cell extracts. Total RNA extraction, cDNA synthesis, and quantitative PCR were performed as previously described (Kunitake et al. 2019). The primers used for qPCR are listed in Additional file 1: Table S2.

## Results

### Effect of *creA*, *pkaA*, and *ganB* deletion on β-xylanase production

**Fig. 1:**
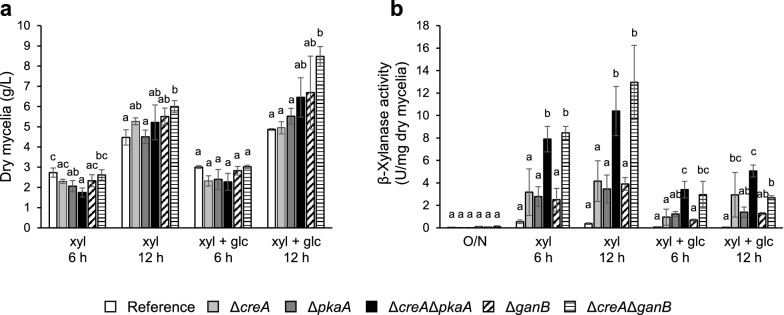
Effect of deletions of *creA*, *pkaA*, and *ganB* on growth and the production of β-xylanase. Precultured strains were further grown in MM containing d-xylose supplemented with or without d-glucose (xyl + glc or xyl) for 6 h and 12 h, and dry mycelial weight (**a**) and total β-xylanase activity in the culture supernatant (**b**) were measured. White bars, the reference strain; light gray bars, Δ*creA*; dark gray bars, Δ*pkaA*; filled bars, Δ*creA*Δ*pkaA*; dashed bars, Δ*ganB*; striped bars, Δ*creA*Δ*ganB*. Error bars indicate the standard deviations of three independent experiments. Different letters indicate a significant difference at *P* < 0.05 by one-way analysis of variance (ANOVA) with Tukey’s post hoc test

The effect of the deletions on growth is shown in Fig. 1a. On d-xylose, all of the strains including the reference strain grew similarly. On d-xylose plus d-glucose, the Δ*creA*Δ*pkaA*, Δ*ganB*, and Δ*creA*Δ*ganB* strains exhibited better growth than the others at 12 h, but the increase in the biomass was less than twofold.

The total β-xylanase activity per milligram dry mycelia produced in the culture supernatants is shown in Fig. 1b. In the overnight precultures with Bacto Peptone as the main carbon source, the β-xylanase activity was extremely low in all the strains, indicating that the deletions did not lead to a significant increase in basal level β-xylanase production. When only d-xylose was used as the carbon source (inducing conditions), all the deletants exhibited a notable increase in the production of β-xylanase activity as compared to the reference strain. The fold increases over the reference strain at 6 h in the Δ*creA*, Δ*pkaA*, and Δ*ganB* strains were 5.9, 5.2, and 4.6, respectively, while the Δ*creA*Δ*pkaA* and Δ*creA*Δ*ganB* strains exhibited 14.6- and 15.7-fold increases. Higher β-xylanase activity in the double deletants compared to the single deletants was also observed at 12 h. As described previously, d-xylose not only functions as a β-xylanase inducer but also as a repressing carbon source in CreA-dependent CCR (de Vries et al. 1999; Orejas et al. 1999, 2001; Mach-Aigner et al. 2012), which accounts for the increase in the Δ*creA* strain. Not only that, the increases in the other deletants indicate that PkaA and GanB are also involved in CCR by d-xylose, and furthermore, the higher activity in the double deletants indicates that CreA and PkaA/GanB independently participate in CCR. Under d-glucose-added conditions (repressing conditions), the production of the β-xylanase activity decreased in all the strains as compared to that in the inducing conditions. Therefore, mechanisms other than those based on CreA and PkaA/GanB are obviously present in CCR of β-xylanase expression. However, tolerance against d-glucose-derived repression was detected in the deletants. While a sevenfold decrease by d-glucose addition was detected in the reference strain at 6 h, the fold decreases in the deletants were 3.3 for Δ*creA*, 2.3 for Δ*pkaA*, 2.3 for Δ*creA*Δ*pkaA*, 3.6 for Δ*ganB*, and 2.9 for Δ*creA*Δ*ganB*. Similar results were also obtained at 12 h.

### Effect of *creA*, *pkaA*, and *ganB* deletion on the transcription of β-xylanase genes

In the β-xylanase productivity measurements described above, a high d-xylose concentration of 1% (67 mM) was applied to support growth. However, such a high concentration caused significant d-xylose-derived repression of the β-xylanase production. To minimize the d-xylose repression, a lower concentration of 3 mM d-xylose, which gave the highest expression of *xlnA* and *xlnC* in the pilot study to determine the optimal d-xylose concentration for induction (data not shown), was used in the transcription analysis of the β-xylanase genes. As a repressing carbon source, 30 mM d-glucose was added under the repressing conditions, which led to significant decrease in the β-xylanase gene expression in the reference strain as described below.

**Fig. 2:**
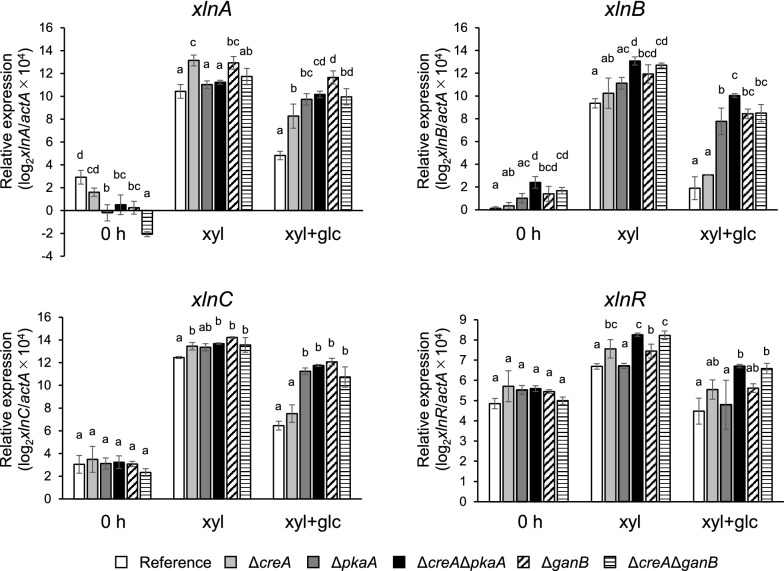
Transcriptional analysis of *xlnA*, *xlnB*, *xlnC*, and *xlnR*. Each strain was cultured under d-xylose-induced conditions (xyl) and under d-xylose plus d-glucose conditions (xyl + glc). Transcription levels of the genes before the addition of the monosaccharides are also included (0 h). The qPCR data were normalized with *actA* encoding γ-actin and are shown on a logarithmic scale of 2 to base (log_2_ (gene/*actA* × 10^4^)). White bars, the reference strain; light gray bars, Δ*creA*; dark gray bars, Δ*pkaA*; filled bars, Δ*creA*Δ*pkaA*; dashed bars, Δ*ganB*; striped bars, Δ*creA*Δ*ganB*. Error bars indicate the standard deviations of three independent experiments. Different letters indicate significant differences at *P* < 0.05 by one-way ANOVA with Tukey’s post hoc test

**Fig. 3:**
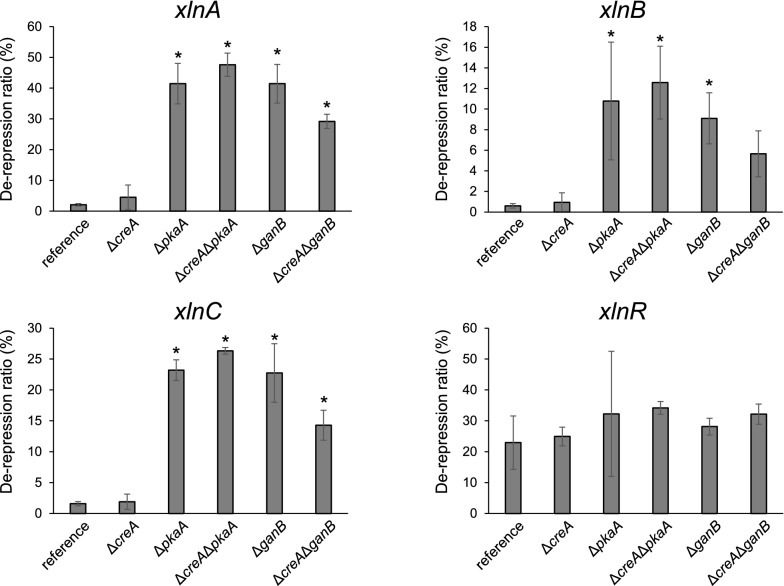
Relative expression levels of the β-xylanase genes on d-xylose plus d-glucose conditions as compared to d-xylose-induced conditions. The de-repression ratio in the vertical axis was calculated by dividing the expression level under the d-xylose plus d-glucose conditions (repressing conditions) by that under the d-xylose conditions (inducing conditions), so that 100% de-repression means the same expression level as that without the repressing carbon source. Asterisks indicate significant differences at *P* < 0.05 by one-way ANOVA with Dunnett’s post hoc test

Increase in expression of β-xylanase genes was observed in all the deletants under d-xylose conditions, and the level of increase differed depending on the deleted gene as well as each xylanase gene (Fig. 2). While the increase confirms that d-xylose has the dual function as an inducer and a repressing carbon source, it also implies that PkaA and GanB are involved in d-xylose repression. d-Glucose addition caused a significant decrease of β-xylanase expression in the reference strain; the expression of the major β-xylanase genes, namely *xlnA*, *xlnB*, and *xlnC*, dropped 48-fold, 168-fold, and 63-fold, respectively, which accounts for 2.1%, 0.60%, and 1.6% of the expression levels in the absence of d-glucose (Fig. 3). In contrast, the decrease in expression levels caused by the d-glucose addition was much smaller in all the deletants except for Δ*creA*. Specifically, the deletants other than Δ*creA* exhibited expression of 29–48% for *xlnA*, 5.7–13% for *xlnB*, and 14–26% for *xlnC* as compared to those in the absence of d-glucose, while the percentages were only 4.5%, 0.94%, and 1.9% in the Δ*creA* strain (Fig. 3). These results imply that the cAMP signaling factors PkaA and GanB play major roles, independently from CreA, in d-glucose-derived CCR of the β-xylanase genes.

However, it should be noted that the ratios were still far below 100%, even in the double deletants, suggesting the presence of a CCR mechanism independent of *creA* as well as *pkaA*/*ganB*. We examined the expression of *xlnR*, which encodes a transcriptional activator of the β-xylanase genes. The expression was repressed by d-glucose in the reference strain, but not completely released by deletion of *creA*, *pkaA*, and *ganB* (Figs. 2, 3).

### Effects of *creA*, *pkaA*, and *ganB* deletions on β-mannanase production

**Fig. 4:**
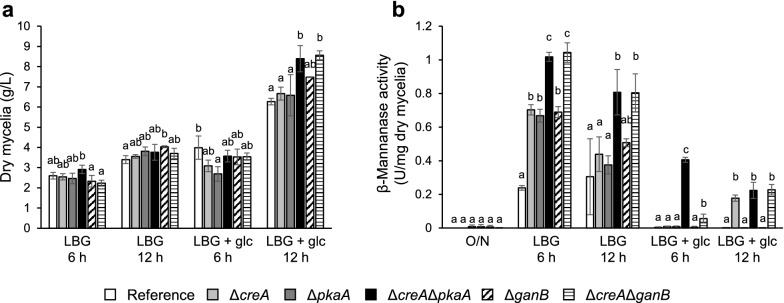
Effect of deletions of *creA*, *pkaA*, and *ganB* on growth and the production of β-mannanase. Precultured strains were further grown in MM containing LBG with or without d-glucose (LBG + glc or LBG) for 6 h and 12 h, and dry mycelial weight (**a**) and total β-mannanase activity in the culture supernatant (**b**) were measured. White bars, the reference strain; light gray bars, Δ*creA*; dark gray bars, Δ*pkaA*; filled bars, Δ*creA*Δ*pkaA*; dashed bars, Δ*ganB*; striped bars, Δ*creA*Δ*ganB*. Error bars indicate the standard deviations of three independent experiments. Different letters indicate significant differences at *P* < 0.05 by one-way ANOVA with Tukey’s post hoc test

To investigate the effect of d-glucose on β-mannanase production in the *creA*, *pkaA*, and *ganB* deletants, precultured mycelia were transferred to MM media containing 0.5% LBG (inducing conditions) or 0.5% LBG plus 1% d-glucose (repressing conditions) and cultivated for 6 or 12 h. Growth of the strains did not differ greatly (Fig. 4a). Although the addition of d-glucose enhanced their growth, reaching 1.7- to 2.3-fold higher biomass at 12 h compared to that without d-glucose, this was simply due to the increase in the amount of the carbon source (Fig. 4a). The double deletants under the repressing conditions grew slightly better (1.3- to 1.4-fold) compared to the reference strain.

β-Mannanase activity in the culture supernatants is shown in Fig. 4b. While β-mannanase activity was extremely low or not detectable in the precultures, the activity of approximately 0.2 to 1.0 and 0.3 to 0.8 U/mg dry mycelia at 6 h and 12 h, respectively, was produced under the inducing conditions with 0.5% LBG. It should be noted that all the deletants displayed increased β-mannanase activity compared to the reference strain, reaching 4.3-fold higher activity in the case of the double deletants at 6 h. The addition of d-glucose caused a significant decrease in the activity. The activity was barely detectable in the reference strain or in the single deletants at 6 h, while the double deletants displayed partial tolerance against the d-glucose-derived decrease. At 12 h, not only the double deletants but also the Δ*creA* strain produced the same level of β-mannanase. These results are nearly identical to those for cellulase production (Kunitake et al. 2019), suggesting that the mechanisms that negatively regulate β-mannanase production are similar to those for cellulase.

### Effects of *creA*, *pkaA*, and *ganB* deletions on the transcription of β-mannanase genes

**Fig. 5:**
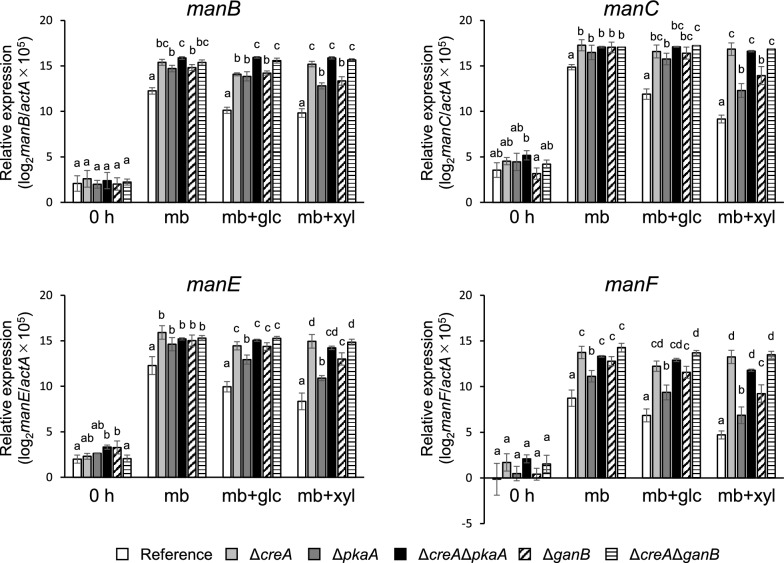
Expression of *manB*, *manC*, *manE*, and *manF*. Each strain was cultured under β-mannobiose-inducing conditions (mb) and d-glucose (mb + glc)- or d-xylose (mb + xyl)-added conditions. Relative expression levels of the genes were normalized with *actA* and are shown on a logarithmic scale (log_2_ (gene/*actA* × 10^5^). White bars, the reference strain; light gray bars, Δ*creA*; dark gray bars, Δ*pkaA*; filled bars, Δ*creA*Δ*pkaA*; dashed bars, Δ*ganB*; striped bars, Δ*creA*Δ*ganB*. Error bars indicate the standard deviations of three independent experiments. Different letters indicate significant differences at *P* < 0.05 by one-way ANOVA with Tukey’s post hoc test

High-molecular-weight substrates such as LBG that induce polysaccharide-degrading enzymes are generally not direct inducers. Small molecules produced by their degradation, typically mono- or di-saccharides, function as the direct inducers that lead to a rapid increase in transcription of the genes encoding the enzymes. In *Aspergillus oryzae*, 1,4-β-mannobiose acts as an inducer of expression of β-mannanase genes (Ogawa et al. 2012). Here, the disaccharide also induced the expression of the β-mannanase genes *manB*, *manC*, *manE*, and *manF* in *A. nidulans*; their transcripts accumulated at 1.5 h after the addition of 3 mM 1,4-β-mannobiose (Fig. 5).

**Fig. 6:**
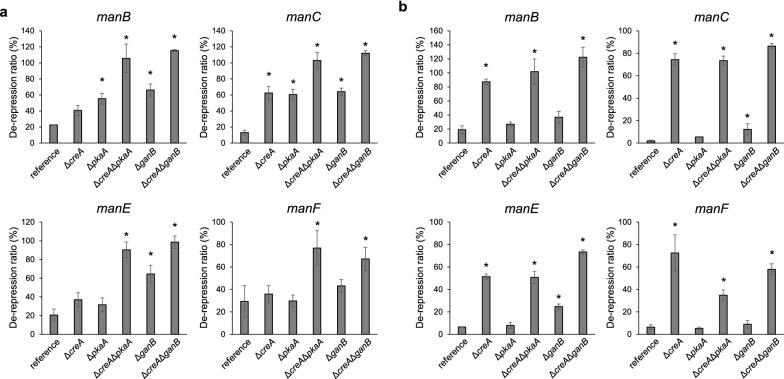
Relative expression levels of the β-mannanase genes on β-mannobiose plus d-glucose (**a**) or d-xylose (**b**) conditions as compared to β-mannobiose-induced conditions. The de-repression ratio in the vertical axis was calculated by dividing the expression level under the repressing conditions by that under the inducing conditions. Asterisks indicate significant differences at *P* < 0.05 by ANOVA with Dunnett’s post hoc test

To evaluate the involvement of the *creA*, *pkaA*, and *ganB* genes in CCR in regulating the β-mannanase genes, the effect of adding d-glucose or d-xylose on the expression of the β-mannanase genes was examined. The addition of d-glucose and d-xylose caused a 3- to 8-fold and a 5- to 50-fold decrease, respectively, in the reference strain (Fig. 5). The expression levels of the β-mannanase genes under such repressed conditions increased in all the deletants as compared to those in the reference strain, and furthermore, it appeared that the contributions of CreA, PkaA, and GanB differed depending on the repressing carbon source (Fig. 6). In the case of d-glucose repression, the expression of β-mannanase genes was partially de-repressed in the *creA*, *pkaA*, and *ganB* deletants. And while the expression levels were 13–29% in the reference strain under the repressed conditions, they increased to 30–66% in the deletants. Furthermore, the repression was nearly fully negated in the double deletants (Δ*creA*Δ*pkaA* and Δ*creA*Δ*ganB*). Although de-repression of the *manF* gene was still partial in the double deletants as a single exception, these results indicated that d-glucose-derived CCR of the β-mannanase genes is regulated by the independent actions of CreA and PkaA/GanB (cAMP signaling). In contrast, CreA predominantly appeared to function in CCR by d-xylose, because de-repression caused by *pkaA* and *ganB* deletion was much weaker than that caused by *creA* deletion (decreased to 2.0–19% in the reference strain, 5.3–37% in Δ*pkaA* or Δ*ganB*, and 51–87% in Δ*creA*). Thus, the contribution of the GanB/PkaA signaling pathway appeared to be very minor in CCR by d-xylose.

## Discussion

In filamentous fungi, CCR mediated by CreA or its orthologs is well known, but the mechanism triggered by CreA is not the only one governing CCR. In a previous study, we reported that PkaA and its upstream factor GanB participate in CreA-independent CCR of the cellulase genes (Kunitake et al. 2019). In this study, we provided evidence for the involvement of PkaA and GanB in CreA-independent CCR of the β-xylanase and β-mannanase genes.

The effects of *pkaA* deletion on β-xylanase gene expression were different depending on the gene (Fig. 2). One possible reason for the discrete dependence on PkaA might be related to the regulation of the β-xylanase genes by the pH-responsive transcription factor PacC. Transcription of *xlnA* is upregulated at alkaline pH, whereas elevated *xlnB* expression occurs at acidic pH (MacCabe et al. 1998). PacC is a phosphoprotein, and its phosphorylation requires PkaA based on phosphoproteomic analysis, although PacC is not a direct target of PkaA (Ribeiro et al. 2019). This report suggests that PkaA indirectly regulates the activity of PacC, and consequently might differently affect the expression of β-xylanase genes.

The expression of the β-xylanase genes is positively regulated by the transcription factor XlnR (Tamayo et al. 2008; van Peij et al. 1998). Based on a previous study, *xlnR* transcription is under the regulation of CreA-dependent CCR, so that CCR of *xlnA* and *xlnB* occurs via CCR of *xlnR* (Tamayo et al. 2008), and in addition, *xlnA* but not *xlnB* is also directly regulated by CreA (Orejas et al. 1999, 2001). Our results in this study are consistent with previous studies: the fold increases in the expression levels in the *creA* deletant compared to those in the reference strain were 1.9 and 2.0 for *xlnR*, 6.4 and 12.5 for *xlnA*, 2.3 and 2.0 for *xlnB*, and 2.0 and 2.2 for *xlnC* (Fig. 2). In contrast to previous reports, however, *xlnR* was repressed in the presence of d-glucose even in Δ*creA*, and complete de-repression of its expression did not occur in any of the deletants (Fig. 3). This indicates that the repression of *xlnR* is mainly caused by uncharacterized mechanisms other than CreA or PkaA/GanB. Possible mechanisms include the factors CreB/CreC and CreD, which have been reported to function CreA-independently in *A. oryzae* (Ichinose et al. 2014, 2018; Tanaka and Gomi 2021; Tanaka et al. 2017, 2018).

Distinct from β-xylanase, the CCR of β-mannanase behaved similarly to that of cellulase; d-glucose repression was mediated by coordinated functions of CreA and PkaA/GanB, while d-xylose repression was mainly mediated by CreA (Figs. 4b, 6) (Kunitake et al. 2019). While the β-mannanase gene expression in the double deletants under the d-glucose-added conditions was almost fully de-repressed (Fig. 6a), β-mannanase activity was still extremely low (Fig. 4b). Although there is no clear explanation for this inconsistency, the major difference between the two analyses is the inducers used: LBG for enzyme production and β-mannobiose for transcription. While LBG needs to be degraded prior to displaying its inducing functions, β-mannobiose itself may directly induce the expression of the genes. Further studies are required to clarify effects of the deletions on direct inducer production from LBG.

In conclusion, this study revealed distinct roles of not only CreA, but also PkaA and GanB in the CCR of β-xylanase and β-mannanase genes, and showed they had different contributions depending on culture conditions and repressing carbon sources. For further understanding of CCR mediated by PkaA and GanB, it is necessary to identify factors that act downstream of PkaA. In addition, other factors involved in CCR must also be studied in detail. Further understanding of the CCR-related factors in concert will facilitate the construction of a completely CCR-free strain by molecular breeding for industrial use.

## Supplementary Information

**Additional file 1: Table S1.** *A. nidulans* strains used in this study. **Table S2.** The primers used for qPCR.

## Data Availability

The data underlying this article will be shared upon reasonable request to the corresponding author.
