# Supplementary material for: cAMP signaling factors regulate carbon catabolite repression of hemicellulase genes in Aspergillus nidulans
Source: AMB Express. 2022 Oct 1;12:126. doi: 10.1186/s13568-022-01467-x (PMC9526778; doi:10.1186/s13568-022-01467-x)
Supplement: Supplementary file 1 — Additional file 1: Table S1. A. nidulans strains used in this study. Table S2. The primers used for qPCR. [file 13568_2022_1467_MOESM1_ESM.docx]

Table S1 *A. nidulans* strains used in this study.

Strain Genotype Reference

ABU *pyrG89 biA1*; *wA3*; *argB2* (Kunitake *et al*. 2019)

Δ*creA* Δ*creA::pyroA pyrG89 biA1*; *wA3*; *argB2*; *pyroA4* (Kunitake *et al*. 2019)

Δ*pkaA* Δ*pkaA::pyroA pyrG89 biA1*; *wA3*; *argB2*; *pyroA4* (Kunitake *et al*. 2019)

Δ*creA*Δ*pkaA* Δ*pkaA* Δ*creA::pyroA pyrG89 biA1*; *wA3*; *argB2*; *pyroA4* (Kunitake *et al*. 2019)

Δ*ganB pyrG89 biA1*; *wA3*; *argB2*; *pyroA4*; Δ*ganB* (Kunitake *et al*. 2019)

Δ*creA*Δ*ganB* Δ*creA pyrG89 biA1*; *wA3*; *argB2*; *pyroA4*; Δ*ganB* (Kunitake *et al*. 2019)

Table S2 The primers used for qPCR.

Gene Name Sequence (5’ to 3’)

*xlnA* QxlnA-F GACTGTGACTACGGCCAACC

QxlnA-R GAAGCCGACCCACTACTCTG

*xlnB* QxlnB-F ACGCAGTTCTGGTCTGTTCG

QxlnB-R TTCCAAGAGTCATGCCAAGC

*xlnC* QxlnC-F ATCACCGTCTGGGGAGTGT

QxlnC-R CATCCTTGGGCTGGTAGTTG

*xlnR* QxlnR-F AGGCTTGTGTCGTTACGCTG

QxlnR-R GCTGCTCACCAAAATCATCTG

*manB* QmanB-F TTTCGCGTGGGAACTAGG

QmanB-R CTTGATGTATGCGCTGACG

*manC* QmanC-F TTGAGGCTGAGCTGAAGTTG

QmanC-R CCACTTGTTCGTCCATTCC

*manE* QmanE-F TGGATGTGTACGCTGACAAC

QmanE-R TCCGGAATATTACCCACCTC

*manF* QmanF-F ATAGCTGGCGACCTTTTCTG

QmanF-R AATCCGCTCCATATGCTCTG

*actA* QactA-F TACTCCGTCTGGATCGGTGG

QactA-R CTTGCGGTGGACGATCGAAG
